# Supplementary material for: Zinc Oxide and Copper Chitosan Composite Films with Antimicrobial Activity
Source: Polymers (Basel). 2021 Nov 9;13(22):3861. doi: 10.3390/polym13223861 (PMC8619498; doi:10.3390/polym13223861)
Supplement: Supplementary file 1 [file polymers-13-03861-s001.zip › polymers-1422978-supplementary.pdf]

Zinc oxide and copper chitosan composite films with antimicrobial activity

Candy del Carmen Gamboa-Solana [1], Martha Gabriela Chuc-Gamboa [1*], Fernando Javier Aguilar-Pérez[1], Juan Valerio Cauich-Rodríguez[2], Rossana Faride Vargas-Coronado[2], David Alejandro Aguilar-Pérez [1], José Rubén Herrera-Atoche[1], Neith Pacheco[3]

[1] Facultad de Odontología, Universidad Autónoma de Yucatán, Calle 61 A #492 A x 90 y Av. Itzáes, Centro., C.P. 97000 Mérida, México; martha.chuc@correo.uady.mx (M.G.C.G.); fernando.aguilar@correo.uady.mx (F.J.A.P), faguilar@correo.uady.mx (F.J.A.A.), jose.herrera@correo.uady.mx (J.R.H.A)

[2] Unidad de Materiales, Centro de Investigación Científica de Yucatán, México. Calle 43 No. 130 x 32 y 34, Colonia Chuburná de Hidalgo, C.P. 97205, Mérida, Yucatán, México; jvcr@cicy.mx

[3] Centro de Investigación y Asistencia en Tecnología y Diseño del Estado de Jalisco, A.C. Parque Científico Tecnológico de Yucatán, km 5.5 carretera, Sierra Papacal-Chuburná, C.P. 97302 Chuburná, México; npacheco@ciatej.mx

SUPPLEMENTARY DATA

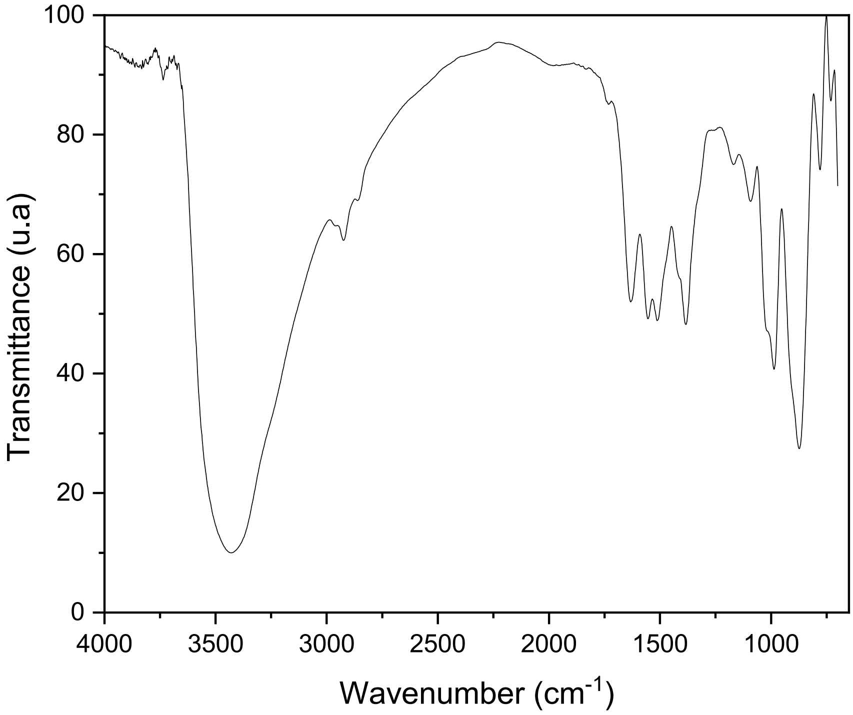

Figure S1. Zn-O FTIR spectra.

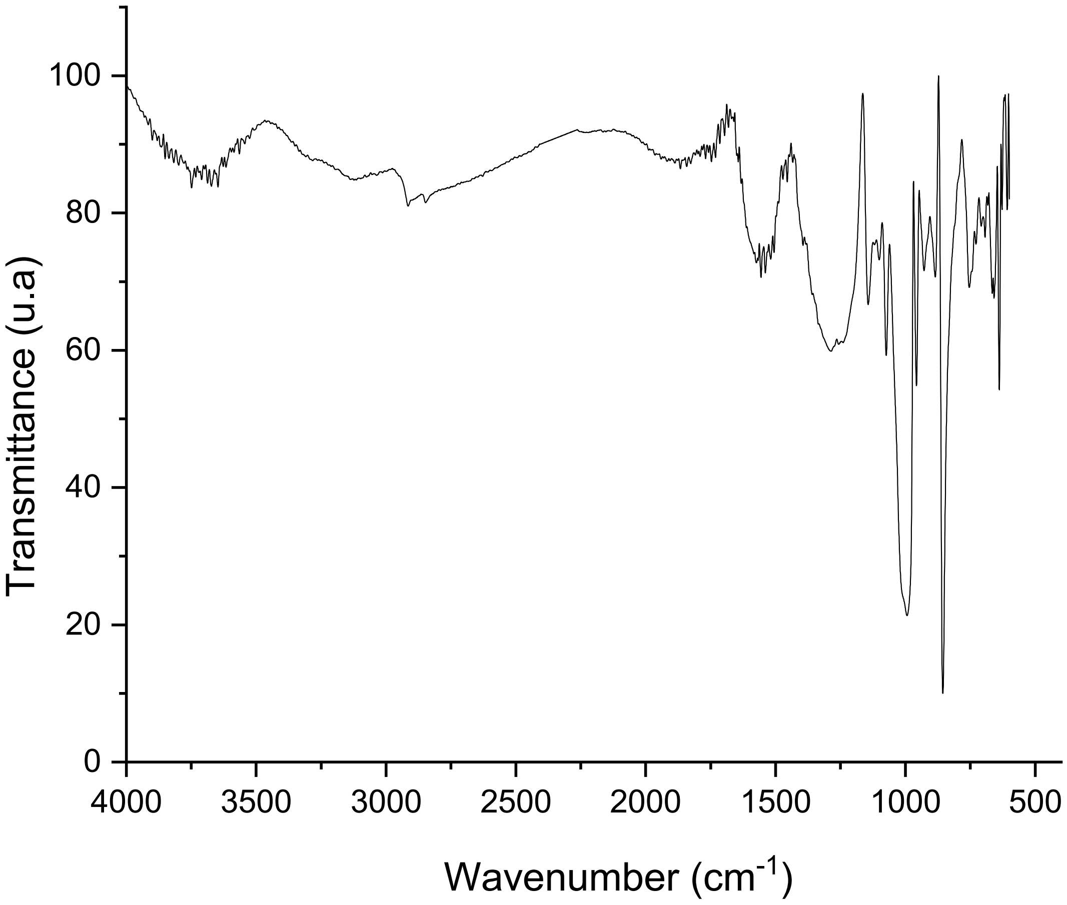

Figure S2. FTIR spectrum of the copper nanoparticles.

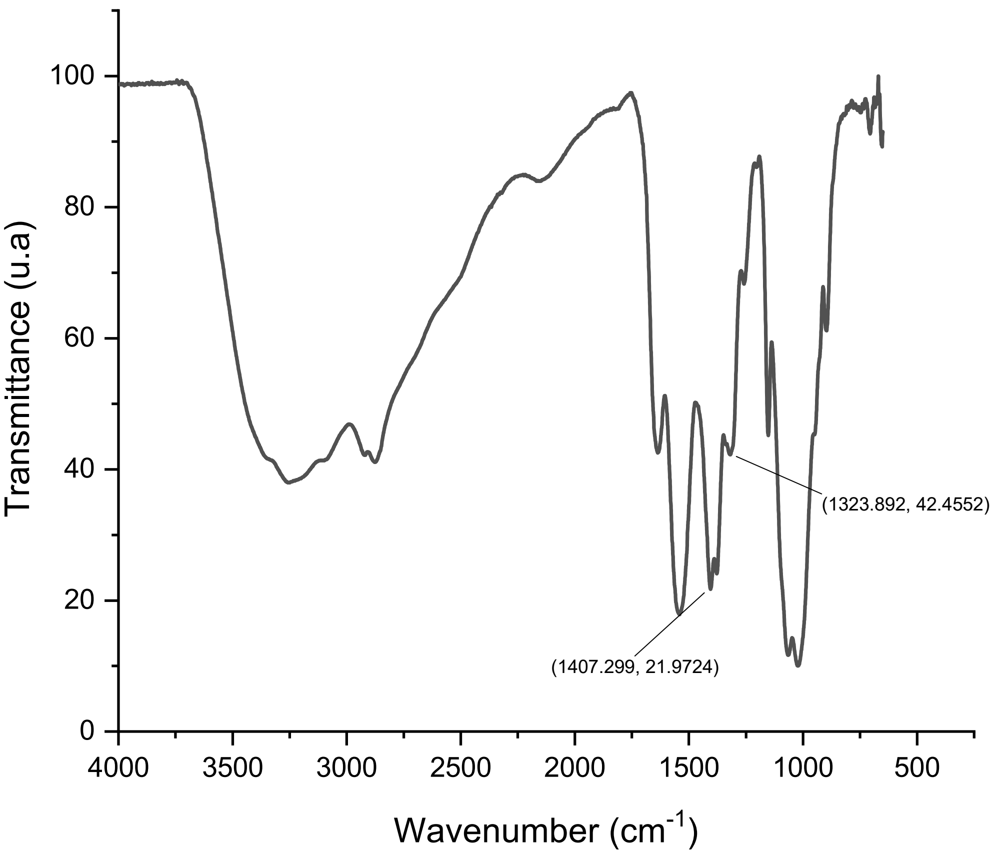

Figure S3. FTIR spectra of pristine chitosan.

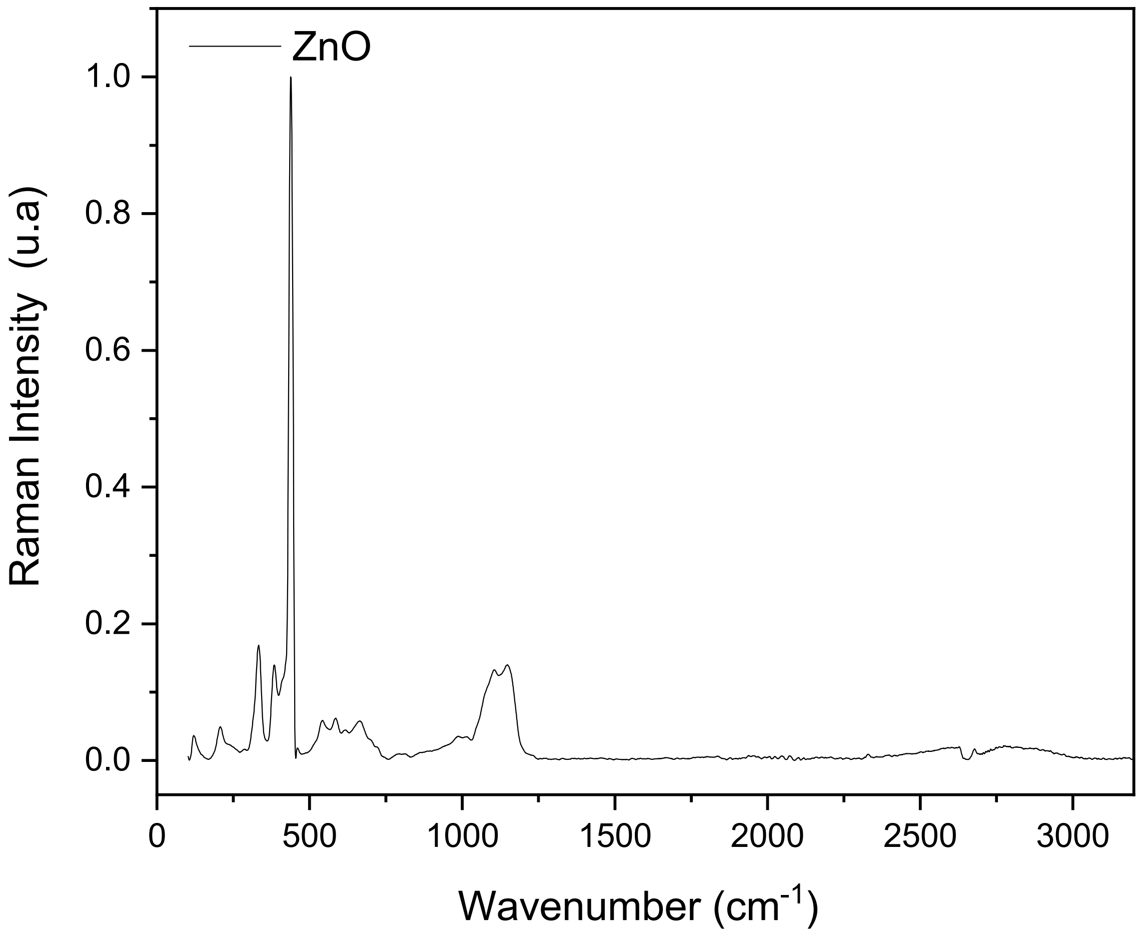

Figure S4. Zn-O Raman spectra.
